# NOX- and ROS-Driven Hypertension in Elastin Insufficiency

**DOI:** 10.1093/function/zqab035

**Published:** 2021-07-09

**Authors:** Gaëtan Gavazzi, Gilles Faury

**Affiliations:** CHU Grenoble Alpes, CNRS UMR5525, Univ. Grenoble Alpes, Clinical Geriatrics Department and GREPI-TIMC-IMAG, 38000 Grenoble, France; Univ. Grenoble Alpes, Inserm U1300, CHU Grenoble Alpes, HP2, 38000 Grenoble, France

## A Perspective on “Inhibition of NOX1 mitigates blood pressure increases in elastin insufficiency”

Williams–Beuren syndrome (WS) is caused by a large deletion in one of the chromosomes 7 (7q11.23), encompassing 26–28 genes, among which is the elastin gene.^1^ Elastin and elastic fibers, essential for large artery structure and function, therefore become deficient and WS features hypertension in half of the patients,^1^ large artery stenoses, and heart disease, while the impact of WS on vessel elasticity varies with the study. Some investigations indicated that WS patients have stiffer large arteries^2^ while other studies showed that these patients have arteries with decreased stiffness,^3^ compared to unaffected persons. While the cardiovascular impact of WS is well known, the molecular pathways leading to altered blood pressure and arterial stiffness in patients with WS or mouse models of the cardiovascular features of WS stays puzzling.

Mice hemizygous for the elastin gene (Eln+/−), the most studied model of the cardiovascular features of WS, are hypertensive, with cardiac hypertrophy, and have stiffer, narrower, and longer large arteries.^4–6^ These vascular features all contribute to elevated vascular resistance (Poiseuille's law), therefore increased postcharge, and could account for increased heart work and blood pressure. In addition, elevated renin levels are found in Eln+/− mice and 40%–50% of WS patients,^7^ which suggests that the renin-angiotensin system could be involved in a basal vessel constriction level and account for the featured hypertension.

**Figure 1.**
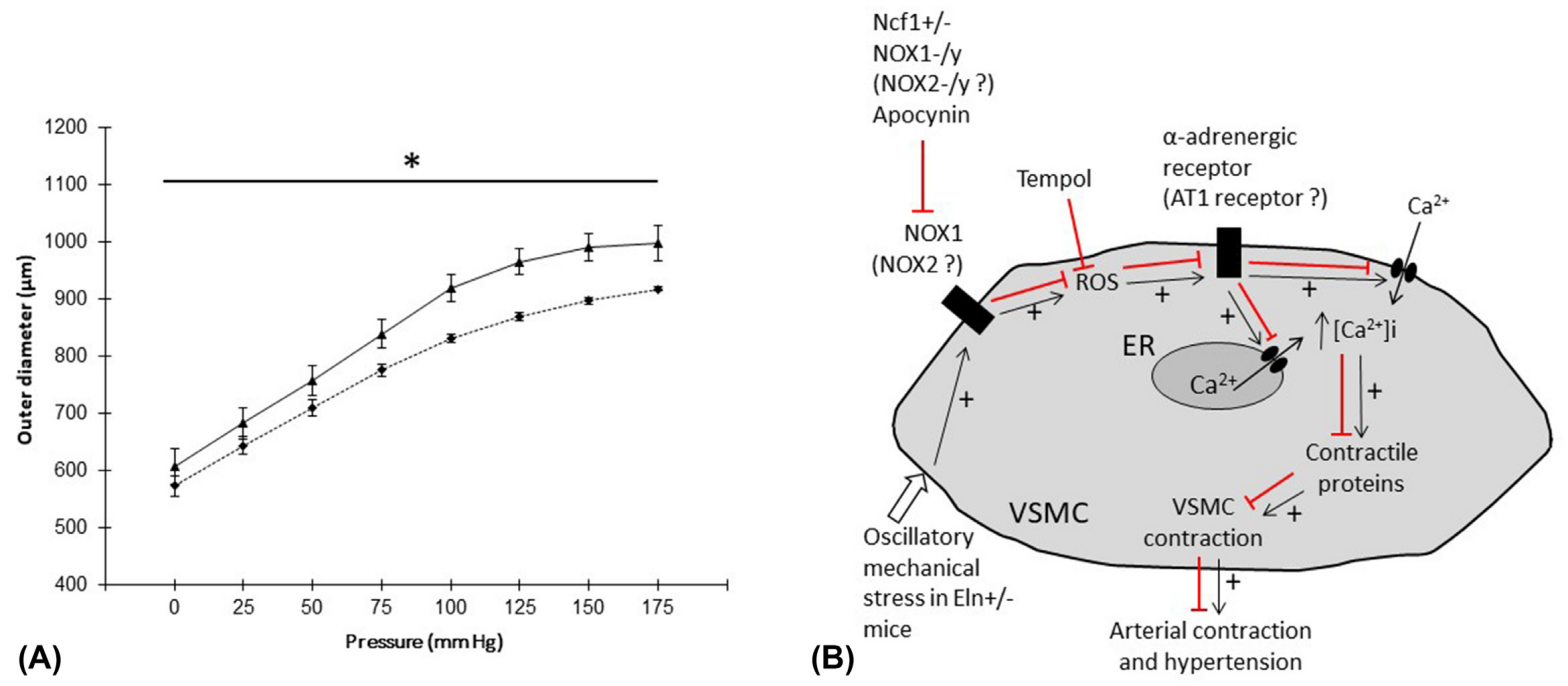
A. Pressure-diameter relationships of cannulated abdominal aortae from adult NOX2-/- mice (gift from Dr. K.H. Krause, Department of Pathology and Immunology, Faculty of Medicine, University of Geneva, Switzerland; dotted line) and corresponding wild-type C57Bl6/J mice (solid line), established by following the experimental procedure previously described.^6^ Values are mean ± SEM. n = 7–8 per group. * General significant difference between NOX2-/- and wild-type mice (2-way ANOVA, *P* ≤ 0.05). B. Proposed schematic summarizing the impact of NOX activity on vascular smooth muscle cell contraction in Eln+/− mice and the effects of treatments, according to Troia et al., and remaining questions. α-adrenergic receptor: phenylephrine (PE) receptor. AT1 receptor: angiotensin II type 1 receptor. VSMC: vascular smooth muscle cell. ER: endoplasmic reticulum. Black arrows: stimulatory pathways. Red arrows: inhibitory pathways.

In the article “Inhibition of NOX1 mitigates blood pressure increases in elastin insufficiency” by Troia et al. published in the present issue of Function,^8^ a new and major molecular cause of hypertension and arterial stiffening in mouse elastin insufficiency is uncovered, that is, reactive oxygen species (ROS). This is in accordance with the suggestions from previous studies which showed that, when the WS deletion included the NCF1 gene, then the patient's blood pressure and arterial stiffness were lower. The NCF1 gene encodes for p47phox, the regulatory subunit of the NADPH oxidase (NOX) complexes NOX1 and NOX2, which generate ROS in the vessel walls, leading to hypertension.^9^ These clinical findings have been confirmed by Troia et al. in Eln+/− mice, shown to have elevated vascular ROS production, and Eln+/−; Ncf1+/− mice, whose arterial cells produce less ROS than those from Eln+/− animals. Because of the specific geometry of the Eln+/− mouse arteries, that is, smaller caliber, longitudinal elongation, and subsequent tortuosity, the blood flow becomes turbulent and exerts a particular oscillatory shear stress on the vessel wall. This results in activation of the aortic endothelial and smooth muscle cells which produce more ROS. By crossing Eln+/− mice with either NOX1-/y or NOX2-/y animals, Troia et al. have also shown that NOX1 is the major contributor to ROS-induced elevation of blood pressure in Eln+/− animals. Indeed, compared to blood pressure in Eln+/− mice, deficiency in NOX1 (Eln+/−; NOX1-/y) induces a strong drop in systolic blood pressure, which is normalized to the level present in Eln+/+ mice, while only a slight trend towards a decrease in blood pressure could be observed in Eln+/−; NOX2-/y mice. Surprizingly, although arterial stiffness was found lower in Eln+/−; Ncf1+/− than in Eln+/− mice at the respective physiologic blood pressure of each group, no difference in the ascending aorta morphology and pressure-diameter relationship could be observed between Eln+/−, Eln+/−; Ncf1+/−, Eln+/−; NOX1-/y, and Eln+/−; NOX2-/y mice, or between Eln+/+, Eln+/+; Ncf1+/−, Eln+/+; NOX1-/y, and Eln+/+; NOX2-/y mice. However, it cannot be excluded that the involvement of NOX2 in the vessel mechanics, very low in the ascending aorta, could be greater in downstream parts of the vascular tree with a higher proportion of endothelial cells. This is suggested by our observation that, compared to wild-type animals, the abdominal aorta of NOX2-deficient mice presents modified pressure-diameter relationships, with narrower vessels in the 0-175 mmHg range and reduced extensibility (% change in diameter, 2-way ANOVA, *P* ≤ 0.05) in the 50–100 mmHg range (Figure 1A). Taken together, the results from Troia et al. suggest that NOX, especially NOX1, deficiency does not modify the ascending aorta developmental process and mechanical properties but, by its lowering effect on blood pressure, rather shifts the working mechanical properties of the arteries to another part of the pressure-diameter curve, where the vessel distensibility between diastolic and systolic pressure is higher.

From a clinical and therapeutic potential point of view, another important finding from Troia et al. relies in the observation that elastin deficiency-induced hypertension could be pharmacologically reversed by chronic—not acute—treatment of Eln+/− mice with a NOX1 and 2 inhibitor, apocynin. Consistantly, the larger elevation of blood pressure in Eln+/− than in Eln+/+ mice, in reponse to high doses of the CMLV-dependent vasoconstrictor phenylephrine (PE), is abolished by NOX1 deficiency (in Eln+/−; NOX1-/y mice) or after acute administration of the ROS scavenger tempol. This suggests that elimination of ROS, immediate (Tempol) or in the long term (chronic apocynin or in Eln+/−; NOX1-/y mice), is sufficient to normalize blood pressure, possibly by reversing the ROS-enhanced production/stability/activity of receptors to vasoconstrictors like PE (Figure 1B). Given the elevated circulating renin level in Eln+/− mice and about half of WS patients, it would be of interest to investigate the impact of ROS-limitating agents like apocynin and tempol on the contraction of the ascending aorta in response to angiotensin II.

The work by Troia et al., by uncovering the role of NOX-generated ROS in WS-related hypertension, opens new therapeutical perspectives. Administration of apocynin to humans has already proved to lower tissular H_2_O_2_ in obese patients.^10^ Acute or chronic treatment with apocynin could therefore be considered for future clinical trials to lower blood pressure in WS patients.

## Data Availability

Datasets are made available by the authors upon request.
